# Adolescent’s time use and skills development: Do cognitive and non-cognitive skills differ?

**DOI:** 10.1371/journal.pone.0271374

**Published:** 2022-07-21

**Authors:** Bareerah Hafeez Hoorani, Jaya Krishnakumar, Paul Anand

**Affiliations:** 1 Nijmegen School of Management, Radboud University, Nijmegen, Netherlands; 2 Faculty of Economics and Management, University of Geneva, Geneva, Switzerland; 3 Faculty of Arts & Social Sciences, Open University, CPNSS, London School of Economics, Oxford University, Oxford, United Kingdom; Monash University Malaysia, MALAYSIA

## Abstract

This study looks at the association of adolescent’s time use on the acquisition of cognitive and non-cognitive (psychological and social) skills, thus contributing to the literature on parental investment and skills development. Specifically, using data relating to adolescent’s time spent on school, study, sleep, and play, we investigate how these relate to cognitive and non-cognitive skills of older Indian children. For cognitive skills we use Peabody Picture Vocabulary Test (PPVT), which is a well-accepted measure of verbal intelligence. For non-cognitive skills, we construct a self-esteem variable using pride and shame questions; and a resilience variable using questions pertaining to whether an adolescent can get external help for coping with problems. Our results suggest that time use in all four types of activity has a positive association in the development of cognitive skills but competing associations when it comes to non-cognitive skills. We conclude that parental inputs into skills development, such as guidance about adolescent’s time-use, are likely to have a differential association depending on the kind of skills being developed.

## Introduction

The success of any society depends highly on its human capital, whose quality depends on the development of the cognitive and non-cognitive skills among adolescents. In this regard, parents are important facilitators of this skills formation and acquisition process. They play a crucial role not only in determining the child’s initial stock of skills, but also in the future accumulation of these skills [1–3].

The aim of our paper is to contribute to the literature on parental investment and skills development, by understanding the effect of parental investment on the cognitive and non-cognitive skills development among adolescent children living in communities of Andhra Pradesh and Telangana in India. This research is important for two reasons. Firstly, India over the past years has seen rapid economic growth, yet still has rising income inequalities [4]. By addressing the important issue of skills formation, this paper can provide important future policy insights to better develop human capital for adolescents who will soon be joining the Indian workforce. Secondly, previous research has proxied parental investment through variables that are specific to the context that the researchers are studying [3, 5–7], and therefore are not easily applicable in different settings, especially in developing countries that face major data limitations. We address this gap by using a novel way to proxy parental investment through time use variables, which we contend can be applied to children of different age groups, living in more varied settings, and belonging to different socio-economic backgrounds. The idea here is that time spent by children in various activities is largely determined by parents even at an adolescent age.

To achieve the objective of our research, we use the Indian Young Lives Dataset for the older cohort (i.e., children were around 12 years old in round two administered in 2006, and around 15 years old in round three administered in 2009) [8]. To measure cognitive skills, we use percentile ranks for Peabody Picture Vocabulary Test (PPVT), whereas for non-cognitive skills we create two variables namely: self-esteem, and resilience. In this research the proxies for parental investment are time use variables that is the time the adolescent spends on playing, sleeping, studying and in school. For our analysis, we employ first difference (FD) estimation as well as instrumental variables (IV) estimation techniques to correct for possible endogeneity.

## Literature review

Research has shown that parents can have considerable influence on adolescents, when it comes to their career choices, attending college, and even subduing risky behaviour such as delinquencies [9–12]. Parents also play an important role when it comes to the development of cognitive and non-cognitive skills development of their children.

In fact, the impact of parental investment on the development of cognitive and non-cognitive skills has been studied extensively by Cunha & Heckman [3, 5, 6]. They unpack this relationship by proxying parental investment using the number of books, special lessons, number of musical instruments, subscription of the newspaper and the number of trips going to the theatre and going to the museum [1]. While their seminal work focuses on younger children in the United States, their work still highlights the influence that parents can have on a child’s cognitive and non-cognitive skills development. Nonetheless, the social and political context of high-income countries varies considerably from that of low- and middle-income countries, and therefore there is a need for more focused research on developing countries. Helmers and Patnam addressed this issue by extending Cunha & Heckman’s work to explore this relationship in the Indian context [7]. They proxy parental investment by using ‘do parent spend more money on child’s education?’, ‘does child work home or outside?’, ‘how many years back was the child made to start formal schooling?’, and ‘how often does the child see the father (daily, weekly, once a month, once a year)’ and the ‘proportion of total household expenditure on following received by the child 1) Education 2) Health 3) Clothing’. However, these studies do not only focus on young children, but the parental investment variables used are also limiting as they are very specific to the context and age group that is being studied [1–3, 5–7, 13].

In recent years, there has been growing interest in researching adolescents. Adolescents are considered as individuals between the ages of 10 until 19 years [14]. They represent the future human capital of a country and can also be important change agents that can bring about social and political changes (e.g., Malala Yousafzai, Greta Thunberg) [15]. Therefore, understanding the influence that parents have on adolescents is particularly helpful and important. In this regard, empirical research on parental control over adolescents shows that this takes place through different pathways (e.g., via rule-setting and decision making, physical punishment, verbal punishment, and/or psychological control) [9–12, 16–18]; and hence variations in outcome can be expected. For example, some studies find that parents tend to have a positive impact on adolescents (e.g. see [19, 20]), while others find that parents may have no influence (e.g., see [21, 22] https://www.ncbi.nlm.nih.gov/pmc/articles/PMC2782391/ - R9). Nonetheless it is clear that parents do seek to influence adolescent offspring on a daily basis via the pathways outlined above and remain a central influence on the social sphere of their child as a result. [11, 17].

The nature of this control, however, can vary considerably across different cultures. For example, parents in Western culture promote independence, and self-expression in children [23]; while parents in Asian cultures promote obedience and respect in children [24]. This difference largely emanates from western culture being higher on individualism, while eastern culture being higher on collectivism [25]. Therefore, for example, safeguarding family harmony is central for parents belonging to collectivist cultures [26] and given that India belongs to a collectivist culture, it is not surprising that parents also tend to exert higher control over adolescents [16]. In fact, in India parents can also exercise significant influence on adolescents, especially on girls, when it comes to marriage (i.e., when they will marry and who they will marry) [27].

Parents can also exert considerable influence on how an adolescent spends his/her free time (i.e., time outside of school [11]. In fact, the way an adolescent spends his/her free time can have both positive and negative development impacts. If free time is used positively, it can allow adolescents to explore their identity, as well as motivation and skills development. However, at the same time adolescents can also use their free time negatively by retorting to risky behaviour such as smoking, drinking, and drug use [11, 17]. In this regard, parents still have influence on how the adolescent engages with his/her available free time, and hence are not passive actors [11].

The above considerations motivate us to take a novel approach and proxy parental investment on adolescents by the number of hours that the adolescent spends on playing, sleeping, studying and at school. In addition, we see time as a more general variable, that can be used for varied settings as well as for different age groups. For example, Cunha & Heckman used the number of trips going to the theatre and museum or number of musical instruments as variables to proxy parental investment [1]. These variables are less applicable to children belonging to a lower socio-economic status and/or living in developing countries. Similarly, ‘proportion of total household expenditure on following received by the child 1) Education 2) Health 3) Clothing’ used by Helmer & Patnam might not be a suitable proxy for parental investment when it comes to adolescents [7]. This is because adolescents might be earning, and consequently the proportion of money going to them by parents might decline, not because of lower parental investment, but because adolescents are now earning. Consequently, a notable limitation of existing scholarship is that it proxies parental investment with variables that are very specific to the context and age group that is being studied. By proxying parental investment using time variables, we believe our approach can be adopted in varying country context, socio-economic backgrounds, as well as different age groups.

Finally, our research distinguishes between cognitive and non-cognitive skills. Cognitive skills such as writing and problem-solving skills can be defined as the level of academic knowledge and understanding, which are usually measured by grades or tests [1, 2, 28, 29]. Non-cognitive skills, as opposed to cognitive skills, comprise mainly attitudes, personality traits and motivational skills of an individual [30]. In the literature, non-cognitive skills are also referred to as soft skills, life skills, character skills, competencies, and personality traits [31]. However the human capital literature is mainly focused on cognitive skills, which is why for a long time in most empirical studies the effect of non-cognitive skills on labour and educational outcomes were ignored [28, 30, 32]. For example, while human capital theory was the first to establish a relationship between the levels of education to future wages [33], it did not address the effect of non-cognitive skills on future income. Today, however, there is a growing literature that highlights the importance of non-cognitive skills on wages and its role in determining future success [29, 30, 32, 34]. In this regard, an important non-cognitive skill is self-esteem, which is defined by how a person evaluates his or her self-worth. High levels or an increase in self-esteem can lead to positive changes [35, 36]. Another important non-cognitive skill is resilience, which is defined as positive adaptation, and measures how well one faces challenges by coming up with coping strategies [31]. Duckworth referred to this trait as grit [37], which can become an important predictor of success [38–42].

Therefore, the aim of this study is to explain the association of parental investment on both cognitive and non-cognitive skills development of Indian adolescents. In this regard, we fill in an existing gap in the current literature by analysing an understudied age group, which are adolescents, belonging to an understudied context of a developing country that is India.

## Methodology

The paper uses data from the Young Lives project coordinated by University of Oxford, which contains a rich and, in some cases, novel set of variables on child development. In this paper, we analyse data for the older Indian cohort. While the constructed panel data for India by Young Lives has good data on cognitive test scores, variables to assess non-cognitive skills have been constructed from responses to a range of relevant questions. Therefore, for cognitive skills, we use Peabody Picture Vocabulary Test (PPVT), a widely accepted test to measure verbal intelligence. Whereas, for non-cognitive skills, we develop variables for self-esteem and resilience. To develop these variables, we use child questionnaires from round two (conducted in 2006) and round three (conducted in 2009). We did not use round one (conducted in 2002) because the child questionnaire is not similar to that of round two and round three [43], and also round one did not have time use variables that are being used as proxies for parental investment. Hence, the final panel data for our Indian sample with all constructed variables comprises of older cohort children (i.e., adolescents) who are around 12 years old in round two and around 15 years old in round three.

### Creating proxy variables for non-cognitive skills

For non-cognitive variables, namely self-esteem and resilience, we create new proxy variables from the Young Lives dataset [8]. We apply confirmatory factor analysis to a set of responses to construct measures of the latent non-cognitive variables, self-esteem, and resilience. Resulting factor scores have been normalized between zero and one so as to bring the scores to the same scale across both rounds.

#### Step 1: Identifying questions from the Young Lives Dataset

To construct our latent variable for self-esteem, questions from the Young Lives Dataset that uses Rosenberg Self-esteem Scale are used [44]. This scale has been tailored to the local context and has also undergone psychometric validation [45]. Therefore, pride and shame questions from the Young Lives are used to construct factor scores for self-esteem. The construction of a latent variable for resilience is more challenging since the dataset had no validated resilience scale. We therefore create this variable by taking guidance from the theoretical literature [31, 37–42], and validate our measure via exploratory and confirmatory factor analysis.

#### Step 2: Conducting a confirmatory factor analysis

Factor analysis is used to identify questions/indicators that are being explained by the latent variable. As the assumption of multivariate normality is violated and the sample is sufficiently large, our estimation uses the Asymptotic Distribution Free (ADF) method. This method of estimation does not need the multivariate normality assumption on both the observed and latent variables and is appropriate in the presence of non-zero kurtosis [46].

To confirm whether the questions/indicators selected for a particular latent variable are correct or not, we conduct the Comparative Fit Index (CFI). The CFI considers the sample size and assumes that not all the latent variables are correlated to each other, and this is compared with the sample covariance matrix. Therefore, to be sure that the variables included do proxy the latent variable, the CFI has to be greater than 0.90 [47]. The last step calculates the factor score for the non-cognitive latent variables, and the score is normalized between zero and one. Table 1 shows all our indicators/questions that we selected for calculating factor scores. The table shows that all factor loadings are highly significant, thus confirming that the latent variable is affecting the selected observed variables (see S1–S4 Tables for more detailed estimation results). Lastly, passing the goodness of fit tests (i.e., the Comparative fit index, see S5 Table) allows us to be confident that factor scores are good proxy variables for non-cognitive skills.

**Table 1:** Factor loadings for round two and round three non-cognitive skills latent constructs.

Observed variable	Latent construct	Round	Standardized coefficients
Is there someone who could help you if you were having a problem with your studies at school?	Resilience	2	0.533***
			(0.163)
Is there someone who could help you if you were worried about something at home?	Resilience	2	0.353*
			(0.200)
Is there someone who could help if you were being teased or bullied by another child?	Resilience	2	0.419***
			(0.130)
Is there someone who could help you if you needed advice about a religious matter?	Resilience	2	0.454***
			(0.07)
Is there someone who could help you if you needed help getting to school or work?	Resilience	2	0. 590***
			(0.122)
Is there someone who would help—if you were having problems at work?	Resilience	3	0.352***
			(0.124)
Is there someone who would help—If you were worried about something at home?	Resilience	3	0.903***
			(0.037)
Is there someone who would help—If you were being teased or bullied by another	Resilience	3	0.399***
			(0.089)
I feel proud to show my friends where I live	Self-esteem	2	0.132**(0.053)
I am ashamed of my clothes	Self-esteem	2	-0.412***
			(0.073)
I am often embarrassed because I do not have the right supplies for school	Self-esteem	2	-0.481***
			(0.061)
I am worried that I don’t have the correct uniform	Self-esteem	2	-0.760***
			(0.082)
I am proud of my shoes/ chappals or of having shoes/ chappals	Self-esteem	3	0.694***
			(0.039)
I am proud of my clothes	Self-esteem	3	0.938***
			(0.026)
I am proud that I have the correct uniform	Self-esteem	3	0.564***
			(0.049)
I feel my clothing is right for all occasions	Self-esteem	3	0.522***
			(0.044)

*** p<0.01

** p<0.05

* p<0.1

### Creating PPVT percentile rank

The Young Lives dataset contains data on the Peabody Picture Vocabulary Test (PPVT) score. PPVT is a widely accepted test to measure verbal intelligence. The test has been conducted in the native language or the language that the adolescent chose to answer in. This test is different from conventional test as it requires the adolescent to identify the picture that corresponds to the word spoken by the interviewer. Hence, the adolescent for this test requires no writing and reading skills. Young Lives provides raw PPVT scores; however, these raw scores can only be compared among the same age group [48]. To avoid the problem of score comparability across rounds, we use percentile ranks [49]. Hence, we generate percentile ranks of PPVT scores for separate rounds, and then combine these percentile ranks to use them in the main regression.

To calculate the percentile rank, the scores are first ranked in an increasing order. Therefore, a higher score should have a higher rank (than a lower score). The percentile rank is then calculated by the following formula for PPVT scores.

$$\frac{rank}{n}x\;100$$ (1)

Here *n* is the total number of adolescents, whereas *rank* is the rank of the score.

#### First difference estimation

We begin the analysis with first difference regression estimations. This provides an initial understanding of the effect of time use on non-cognitive and cognitive skills. The model also includes the round, regional controls, parental controls, adolescent controls, and household controls (see S6 Table).

Using first difference estimation provides a way to account for time-invariant unobserved heterogeneities, which might derive from school, family, parents, and the community. Our main regression equation is the following:

$$y_{it}=\delta +{T\prime }_{it}\beta_{1}+{x\prime }_{it}\beta_{2}+R_{t}+\mu_{i}+v_{it}$$ (2)

*y*_it_ represents the dependent variable (the cognitive or non-cognitive skills score). Here *i* represents the adolescent in our panel data, whereas *t* represents the two separate rounds (rounds 2 and 3). δ is the intercept of our model. *T’*_*it*_ represents four-dimensional row vectors of our time use variables that are time variant, and β_1_ represents four-dimensional column vectors of parameters. Lastly, *x’*_*it*_ represents time variant controls, and β_2_ are column vectors of parameters for our controls. R_t_ represents a dummy variable for round three, where round three (i.e., when t = 3) equals to 1 and round two (i.e., when t = 2) equals to 0. μ_*i*_ represents the unobserved heterogeneities. Lastly, *v*_*it*_ represents the error term in our model.

#### Instrumental variable estimation

For the cognitive equation, there might be a potential endogeneity issue with time spent by the adolescent studying outside school, and time spent playing. One concern is that if test scores are down, parents might want their child to spend more time studying and less time playing. Lower or higher test scores might influence the time that an adolescent spends on study and play. Thus, there is reason to believe that the covariance between time spent (studying and play) and the error term is not zero. At the same time, we assume that time spent sleeping and at school are exogenous: even if test scores are to go up or down, school time should not change as all adolescents are supposed to spend a fixed number of hours in school. Furthermore, parents will likely not change the sleep time of their child if test scores were to go up and down. Arguably, it is plausible that the two time use variables parents might want to change are those of study and play. This motivates us to conduct a two stage least squares.

For the endogenous variables, play time and study time, we select instruments that satisfy exogeneity and relevance. Exogeneity refers to when the chosen instrument does not correlate with the error term, while by relevance we mean that the instrument should correlate with the endogenous variable, but not with the dependent variable (i.e., PPVT score). To select these instruments, we use theoretical and/or statistical reasons.

We contend that a caregiver’s attitude towards wanting a child would have an influence on the time that the parent lets the adolescent spend on play and studying. However, this attitude would not have a direct effect on the adolescent’s cognitive skills. This is because the reasons for wanting children would have taken shape before the adolescent was born and is unlikely to change much afterwards. Hence attitude towards having a child can be assumed to be exogenous with respect to the adolescent’s outcome (cognitive and non-cognitive skills). In this regard, round two of the Young Lives Dataset has a unique section ‘Caregiver Perceptions and Attitudes’. We use information in this section to create our parental attitude variable, as the primary caregiver for 96% of adolescents in our sample is the parent. This section contains the following five items, which reports the caregiver’s reasons for wanting children, rating them from ‘not important at all’ to ‘very important’:

1. Because having children increases your sense of responsibility and helps you to develop.
2. Because it is fun to have young children around the house.
3. Because of the pleasure you get from watching your children grow.
4. Because of the special feeling of love that develops between a parent and a child.
5. Because raising children helps you to learn about life and yourself.

We use the above items to create a simple arithmetic mean index, which is normalized between zero and one. Since, we also assume that attitudes do not change over time, we therefore do not account for them in our first estimation regressions. We also select a few additional variables as good candidates for valid instruments. For instance, the household size may influence how the adolescent spends his/her time, but this would not have a direct association with PPVT scores. This would also be true regarding the number of children in the household. The geographical location of rural and urban areas may also have an indirect effect on PPVT scores. For example, parents in rural areas are less educated than parents in urban areas. Therefore, an adolescent in the rural area might have to spend more hours studying, as parents can offer less help than an adolescent in the urban area who may seek more help from his/her parents. Time spent travelling to school might be another variable that would have a direct effect on time spent playing and time spent studying but will not have a direct association with PPVT scores. Problem with reading is also a relevant instrument. This is because it may influence study time but will not affect PPVT scores.

Therefore, for our PPVT equation, we use parental attitude, household size, number of children, urban/rural, travel time to school, and problem with reading as our instruments for play time and study time. These instruments pass both the exogeneity and overidentification restrictions test. For the exogeneity test, the null hypothesis is that the time use variables, play, and study, are exogenous. Since we reject the null hypothesis (see Table 2), time use variables for play and study should be treated as endogenous variables.

**Table 2:** Exogeneity test for PPVT scores.

Endogenous Variable	
Hours Spent playing & Hours spent studying	Robust scores *χ*^2^ = 10.2719 (p = 0.0059)
	Robust regression F (2,809) = 5.25983 (p = 0.0054)

Wooldridge’s score test and F-statistic

Over-identification restrictions tests the validity of the instruments. For the instruments to be valid, the statistic should not be significant. We report below Sargan’s and Basmann’s χ^2^ tests, and since the *p-value* is high we can conclude that the selected instruments are valid.

                χ2 = 6.14607 (p = 0.1885).

## Findings

### Descriptive statistics

We follow the same set of 535 adolescents for two rounds. Therefore, the total number of observations present in our pooled dataset are 1070 for both rounds. The dataset has 89.16% Hindus, 48.60% male, with 74.77% attending public schools, and 79.81% belonging to rural areas. Moreover, 32.71% of adolescents belong to Scheduled Caste (SC) and Scheduled Tribe (ST), a group that is considered among the most disadvantaged socio-economic groups in India [50]. Compared to the 2011 census conducted for the state of Andhra Pradesh, there are 66.89% adolescents living in rural areas, 25.82% belonging to the Scheduled Caste (SC) and Scheduled Tribe (ST), and 50.58% adolescents are male [51]. As such our dataset overrepresents adolescents from rural areas and Scheduled Caste (SC) and Scheduled Tribe (ST).

For the cognitive variable PPVT score, we find that the mean as well as standard deviation does not change from round two to round three. In fact, the t-test of the difference in means between the two rounds is not significant. For non-cognitive factor scores, we find that both resilience and self-esteem have a mean higher than 0.5 (see Table 3). For the factor scores of our non-cognitive variables, we find that not only for the pooled data, but also for the two individual rounds that resilience has a higher mean than self-esteem. We also find that the means for both resilience and self-esteem decline over time, and the t-test of the difference in means between the two rounds is significant for both non-cognitive variables. However, the decline for resilience is smaller than the decline for self-esteem.

**Table 3:** Descriptive statistics.

	Round two (R2)				Round three (R3)				Panel				
	Mean	SD	Min	Max	Mean	SD	Min	Max	Mean	SD	Min	Max	Mean Difference (R2-R3)
**Dependent variables**													
**PPVT scores (cognitive)**	50.09	28.89	0.19	99.9	50.09	28.89	0.17	100	50.09	28.88	0.19	100	0
**Self-esteem (non-cognitive)**	0.86	0.23	0	1	0.68	0.24	0	1	0.77	0.25	0	1	0.177***
**Resilience (non-cognitive)**	0.98	0.08	0	1	0.95	0.18	0	1	0.97	0.15	0	1	0.028***
**Independent variables**													
**Time spent playing (hours)**	3.94	1.95	1	12	3.94	1.83	1	12	3.94	1.89	1	12	0
**Time spent in school (hours)**	6.85	1.07	4	10	8.19	0.96	4	11	7.49	1.22	4	11	-1.341***
**Time spent studying (hours)**	2.26	1.23	1	7	2.63	1.13	1	7	2.44	1.19	1	7	-0.3714***
**Time spent sleeping (hours)**	9.02	0.84	6	11	8.15	0.85	6	11	8.59	0.95	6	11	0.866***

T-test of the difference in means significant at

*** p<0.01

** p<0.05

* p<0.1

For the key independent variables, we see that the mean for the number of hours spent on playing for both rounds is 3.94 hours. Therefore, the mean for this variable does not change from the second round to the third. In fact, the t-test of the difference in means is also not significant. However, there is a slight decline in the standard deviation from 1.95 hours for round two to 1.83 hours for round three. This might be because adolescents either are devoting more time to studying or to other activities. We, however, do see an increase in the mean for the number of hours spent studying, where the mean for round two is 2.26 hours to 2.63 in round three. This looks intuitive, since now adolescents are studying in a higher grade and will need to spend more time on their studies. Moreover, we expect the homework load to also increase as the adolescent moves to a higher grade, which might be affecting the increase in time spent studying. In this regard, we also find that the t-test of the difference in means between the two rounds for the time spent studying is significant. Hours spent on sleep declines from 9.02 hours in round two to 8.15 hours in round three. This decline can be attributed to time being spent on other activities. In this regard, we also find that the t-test of the difference in means between the two rounds for the time spent sleeping to be significant. We also see the average number of hours spent in school increase from 6.85 hours in round two to 8.19 hours in round three. This increase might be because in round three adolescents are 15 years old, and hence are in a higher grade in which school time is longer. We also find that the t-test of the difference in means between the two rounds for the time spent in school is significant.

### Results of first difference estimation & instrumental variable estimation

We want to point out here that we discuss our findings from the first difference and instrumental variable estimations in terms of associations rather than causality. We do this as a precaution to acknowledge that we are limited by the available variables in our dataset as well as in the choice of instruments, and hence would like to take a minimum risk approach in making causal claims.

For self-esteem time use variables for play, school and study are all negatively significant. Therefore, an extra hour of time spent on play will decrease on average the association with factor scores for self-esteem by 0.0219. This might be because of unsupervised time play, where adolescents might be getting bullied from each other thus hurting their self-esteem. Furthermore, one extra hour spent in school on average decreases the association with factor scores for self-esteem by 0.0236. This result is particularly alarming, because we would expect schools to play an important role in developing self-esteem of an adolescent, however as our result shows that this is not the case. One reason for this negative relationship is the widespread use of corporal punishment in the state of Andhra Pradesh [18]. Hence this result suggests that the quality of education in the region needs to be improved. Lastly, an extra hour spent on studying decreases the association with factor scores for self-esteem by an average of 0.0354, which is highly significant. This result is also concerning and could arise for several reasons, which we discuss in detail in the discussion section of the paper.

The factor scores for resilience are primarily determined by whether an adolescent could seek help when facing a problem. Under the first difference regression all time use variables are positively significant on resilience that is time spent sleeping, playing, studying and at school (see Table 4). For the time use variable play, we observe that an extra hour of play on average increases association with resilience by 0.0136. This is intuitive since while playing, especially in group, adolescents do confront problems and they must find ways to cope with these problems by asking help from peers or from an adult. Furthermore, an extra hour of study time increases association with resilience by an average of 0.015. This shows that the more time an adolescent spends studying, the chances of facing a problem in understanding schoolwork increases. Therefore, this challenge allows them to seek help from adults around them. However, this association is significant at *p-value* that is less than 10% but greater than 5%. Similarly, an extra hour spent in school also increases association with resilience by an average of 0.0124, but again the association here is significant at a *p-value* that is less than 10% but greater than 5%. Interestingly time use variable for sleep is also significant thus alluding to the role that adequate sleep plays not only in the health of an adolescent [52], but also in the development of non-cognitive skills such as resilience.

**Table 4:** First difference and instrumental variable estimations.

	*First Difference Estimates*			*Instrumental Variable Estimation*
VARIABLES	Self Esteem	Resilience	PPVT	PPVT
Time spent sleeping	0.0113	0.0286**	2.629**	11.26**
	(0.0151)	(0.0116)	(1.195)	(4.890)
Time spent playing	-0.0219**	0.0136**	2.659***	19.88***
	(0.0093)	(0.0056)	(0.7976)	(6.889)
Time spent at school	-0.0236**	0.0124*	1.379	18.67***
	(0.0110)	(0.0071)	(1.095)	(6.134)
Time spent studying	-0.0354**	0.015*	3.092**	19.46**
	(0.0143)	(0.0072)	(1.362)	(8.468)
Regional controls	*Included*	*Included*	*Included*	*Included*
Parental controls	*Included*	*Included*	*Included*	*Included*
Adolescent controls	*Included*	*Included*	*Included*	*Included*
Household controls	*Included*	*Included*	*Included*	*Included*
Round	*Included*	*Included*	*Included*	*Included*
Constant	0.377	0.438	-324.7***	-337.0***
	(0.979)	(0.343)	(99.66)	(118.7)

Robust standard errors in parentheses

*** p<0.01

** p<0.05

* p<0.1

For, PPVT score that measures cognitive skills we find that after controlling for time invariant heterogeneities (e.g., unobserved family, parental and community characteristics) number of hours spent playing, studying, and sleeping are significant. One hour of play increases on average the association with percentile rank of PPVT score by 2.659, whereas for one extra hour spent studying the association with percentile PPVT rank on average increases by 3.092, and finally for one extra hour of sleep increases the association with percentile PPVT rank by 2.629. However, we find it counterintuitive that time spent at school is not significant. We believe this is because of the endogeneity issue arising from time spent playing. This, therefore, motivates us to conduct an instrumental variable estimation. After controlling for possible endogeneity, we find that all our time use variable associations are positively significant. Furthermore, the magnitude of our coefficients has increased considerably, which shows that the problem of endogeneity might have been the chief reason for low values of time use coefficients in the first difference estimation. Therefore, with IV estimation, one hour of extra play, study, school, and sleep on average increases the association with percentile rank of PPVT scores by 19.88, 19.46, 18.67 and 11.26 respectively. Therefore, our results show that for PPVT scores time use variables for sleep, play, school, and study are important for the development of verbal intelligence. Time use variables for school and study being significant goes with our intuition, as both school and study should increase verbal intelligence. However, our result also highlights the importance of sleep and play. Our results show that play time (or physical activities) even for an adolescent is important, a result that has been corroborated in the literature [53]. Furthermore, sleep is also important for verbal intelligence. This might be because sleep plays an important role in physical and cognitive development, in which inadequate sleep can lead to cognitive deficits that can affect attention and memory [54] (for more detailed results check S7–S10 Tables).

To check for the robustness of our results, we introduce two shock variables in our regression, which are illness of mother and illness of father. Upon running our first difference regression, we find no significant changes in our results (see Table 5, and for more detailed results check S11–S14 Tables), thereby leading to the conclusion that our results are robust.

**Table 5:** First difference and instrumental variable estimations with shock variables.

	*First Difference Estimation*			*Instrumental Variable Estimation*
VARIABLES	Self Esteem	Resilience	PPVT	PPVT
Time spent sleeping	0.0123	0.0279**	2.6189**	11.40**
	(0.0153)	(0.0115)	(1.2041)	(4.907)
Time spent playing	-0.0220**	0.0136**	2.6573***	20.00***
	(0.0091)	(0.0056)	(0.8021)	(6.908)
Time spent at school	-0.0233*	0.0122	1.3800	18.83***
	(0.0110)	(0.007)	(1.1022)	(6.166)
Time spent studying	-0.0362**	0.0154**	3.0814**	19.79**
	(0.0142)	(0.007)	(1.3543)	(8.508)
Shock 1: Illness of mother	-0.0417	0.0311	0.2184	1.197
	(0.0323)	(0.0212)	(3.0766)	(4.543)
Shock 2: Illness of father	0.0148	-0.0118	-1.377	-0.317
	(0.0468)	(0.0174)	(3.6997)	(4.367)
Regional controls	*Included*	*Included*	*Included*	*Included*
Parental controls	*Included*	*Included*	*Included*	*Included*
Adolescent controls	*Included*	*Included*	*Included*	*Included*
Household controls	*Included*	*Included*	*Included*	*Included*
Round	*Included*	*Included*	*Included*	*Included*
Constant	0.482	0.361	-329.5***	- 340.4 ***
	(0.978)	(0.343)	(100.2)	(119.3)

Robust standard errors in parentheses

*** p<0.01

** p<0.05

* p<0.1

## Discussion

The aim of our paper is to investigate skills formation of Indian adolescents. More specifically, we investigate the association of parental investment on the cognitive and non-cognitive skills development of Indian adolescents. We find that there is an association between parental investment on adolescents’ cognitive and non-cognitive skills. More importantly, we also find that this association on the non-cognitive skills can be both negative (i.e., for self-esteem) as well as positive (i.e., for resilience). These competing associations, we contend, highlights the importance that quality of time spent playing, studying, in school and sleeping can potentially have on the non-cognitive skills development among Indian adolescents. Considering these insights, we see our paper contributing to the much-needed policy discussion around skills development of adolescent children in India that suggests time spent on an activity while being important is not an end in itself. In fact, the quality of how this time is spent needs to be considered as well. In the subsequent sub-sections, we discuss the implication of our results in more detail along with possible policy recommendations that can help address the issues that we unpack in this study.

### Improving quality of play time

While much of the literature has focused on early years of childhood, our result corroborates the conclusions of existing literature that play is important for both cognitive and non-cognitive skills development [55, 56]. An important insight from our study is that the time an Indian adolescent spends on play has a positive association on cognitive skills, however for non-cognitive skills, we find that it is negatively associated with self-esteem and positively associated with resilience. Regarding the positive association of play with cognitive skills, we see that our findings corroborate existing insights from the literature [56]. Nonetheless research on play has focused mostly on early childhood, and as such studies on the importance of play for adolescents is absent. This is surprising given that the United Nations High Commission for Human Rights considers it to be a right for every child (in which a child is defined as an individual below the age of 18 years) [57].

Despite its acknowledged importance there is a dearth of research that can explain the association of play on adolescents. As our study shows, play is still very important for adolescents’ cognitive and non-cognitive skills development, yet not all forms of non-cognitive skills benefit from play. A limitation here is that except for the number of hours an adolescent spends on playing, we do not know from the available data the quality of play. Hence from here on, we are only speculating possible explanations, and in this regard more research is needed on the quality of play for adolescents in India.

We hypothesize that the reason for the negative association between play and self-esteem is that adolescents in our dataset are unable to engage in quality playtime. This could be due to various reasons, one being that playtime is unsupervised, which increases the chances of an adolescent being bullied thereby decreasing self-esteem. However, one could also argue that supervised playtime for adolescents may not be possible, as adolescents may want more autonomy from their parents. As such from a policy standpoint, one possible solution could be that regional state provides safe access to playgrounds. In fact, safe playgrounds that provide an inclusive and visible physical space, can deter unsocial behaviour and bullying [58]. Unfortunately, a lack of access to safe playgrounds is an issue, not just for the state of Andhra Pradesh and Telangana, but across India. Moreover, this lack of access gets exacerbated when it comes to impoverished communities [59].This is not surprising, because there are no policies in India that directly addresses the issue of play and more specifically quality play [60]. In fact, a news article in the Times of India reported that Ludhiana (a city in India) has about 800 parks, but none of them have a playground for children [61].

In this regard both State and Central governments need to address the issue of play not only for young children but for adolescents as well. More importantly there needs to be policies that can address quality play, because a lack of quality play may end up harming the self-esteem of Indian adolescents that can lead to several mental health problems such as anxiety and depression [62].

### Improving quality of school time

From a policy standpoint the Government of India has made education one of its priorities, as it sees education as an important aspect for developing its human capital [63]. For example, India’s constitution states that it will provide free education to children up to fourteen years of age [64]; and the Right of Children to Free and Compulsory Education (RTE) Act (passed in 2009) states that children from 6 to 14 years of age should have access to good quality education [65]. Hence, school enrolment in India has seen significant increase for both secondary and higher secondary education [63, 66].

The fact that we see a positive association between school time and PPVT score shows that efforts from the Indian Government on education are paying off in terms of cognitive skills development. However, we also find that time spent at school has a negative association on self-esteem, and a weak positive association with resilience, which disappears when we introduce shock variables pertaining to the illness of the mother and father for our robustness check. These results highlight, as is the case with play time, the importance of quality of schooling. In fact the issue regarding quality of schools, in spite of the governmental efforts, has been well documented, see e.g. [66–68].

In this regard, we would like to highlight three aspects of low-quality schooling that might lead to this negative association with self-esteem. First, low quality schooling represents varied levels of educational achievements between different income groups [69]. For example, Teach for India on their website indicates that children in impoverished communities are behind their actual grade level, and therefore “more than 50% of students in Grade 5 cannot read a Grade 2 text or solve a simple subtraction problem” [70]. If the grade disparity is so high, we can naturally conclude that in all likeness adolescents in our dataset face the same issue. As a result, an adolescent’s time at school may be hurting his/her sense of self-esteem, as he/she might feel that s/he is not intelligent. Second, corporal punishment is also very rampant in schools in India, as well as in Andhra Pradesh, in which teachers see it as an acceptable disciplinary method; and this conviction is stronger for teachers catering to schools in impoverished communities [18, 71]. This is in spite of the fact that the Indian government has a legislation, the Right of Children to Free and Compulsory Education Act (2009), which prohibits corporal punishment in both private and public schools [65]. The negative effects of corporal punishment on a child’s mental and physical health is well accepted [18, 72, 73]. Considering this we contend that the negative association between time spent at school and self-esteem might be emanating from the widespread acceptance and use of corporal punishment in Indian schools [18, 71, 74]. Another aspect to consider when thinking of the quality of schools is bullying. In fact, the Young Lives Dataset has been collecting qualitative data (e.g., in-depth interviews) to gauge a better understanding of the child/ adolescent’s point of view. While this data is not publicly accessible due to confidentiality concerns, a report released by the Young Lives that analysed this qualitative data suggests that adolescents in India experience bullying [75]. Moreover, bullying represents a wider issue at the school level, where adolescents replicate the teacher’s use of corporal punishment on their peers [18, 75]. This might also explain the negative association that we witness between school time and self-esteem.

Before we move to policy suggestions and recommendations to tackle this issue, it is worth noting that while both playtime and school time share a negative association with self-esteem, the former however has a positive association with resilience and the latter has a weak relationship that disappeared during robustness checks. It is important to understand why there is this difference between the two-time variables. One possible explanation could be that during playtime an adolescent may have more control over his/her situation as they can get away from a toxic situation and might receive the needed help from someone. For school time, however, external help might be more difficult to receive. For example, parents in most cases would condone the use of corporal punishment by a teacher at school [18]; so even if an adolescent would complain to parents, it is unlikely he/she will receive any help. Similarly, if the adolescent wants to change schools, he/she may be unable to do so, as access to good private schools comes with higher tuition fees, thus disabling this transition.

Consequently, what our study shows is that while efforts from both the Central and State government is paying off when it comes to cognitive skills development, it still needs to introduce policies that can also develop non-cognitive skills of adolescents (and even for younger children). The government (both at the state and federal level) can begin by addressing the use of corporal punishment as a form of disciplinary method in schools. This can be done using a two-pronged approach, by first actively raising awareness in schools about the negative effects of corporal punishment on adolescents. Second, by providing teachers adequate training on classroom management to deter them from retorting to corporal punishment as a form of disciplining students. These recommendations could be important starting points in making schools a safe space for adolescents, thereby enabling these adolescents to reach their full potential.

### Improving quality of study time and increasing awareness on the importance of sleep

Our study shows that time spent studying has a positive association with PPVT scores and resilience but has a negative association with self-esteem. Study time should intuitively increase PPVT scores, and this is what our result also shows. However, we find again competing associations with non-cognitive skills (i.e., self-esteem and resilience). Again, the same reasoning discussed in the previous two sub-sections can be postulated in which the quality of study time needs to be improved.

Therefore, a possible explanation is that adolescents when they seek external help, they will get it during their study time, for example a family member (e.g., siblings or parents) may agree to help the adolescent in their study. This therefore explains the positive association between study time and resilience, because the more the adolescent studies, the more he/she may need external help which he/she is likely to get. At the same time, it is possible that when they seek for help, they may also receive negative remarks (i.e., verbal abuse) on their capabilities if they do not perform well on their studies, and even corporal punishment. This might explain the negative association between study time and self-esteem.

Another possible reason for the negative association between study time and self-esteem might be that longer study hours may reflect the inability of the adolescent to cope with the high expectations from their entourage [69]. It also could be that parents may justify extra hours of study by telling the adolescent that it will improve their circumstances in the future. This could make the adolescent more aware of his or her disadvantaged position, as such hurting his/her self-esteem.

On a policy level, there needs be more concerted effort of raising awareness of the downside of corporal punishment and verbal abuse on the adolescent’s development. However, concrete policies to address this issue is difficult, and can only happen if corporal punishment stops in schools first, who can then make parents accountable for the violence (both verbal and physical) that they use on the adolescent at home. As a first step, increasing awareness around this issue by adopting zero tolerance on any form of violence against adolescents and younger children, can bring about the much-needed change of mindset at the societal level around this issue.

Finally regarding sleep, research has also shown that sleep deprivation can negatively affect academic performance among adolescents, and can lead to behavioural problems [76–78]. Consequently, adequate sleep improves academic performance as learning, attention and memory improves [76, 77]. Our study corroborates this finding because sleep has a positive association with PPVT scores. Another interesting insight that emerges is its positive association with resilience. This result makes the case that benefits of sleep extend beyond cognitive skills to non-cognitive skills. However, sleep is still an under researched topic for adolescents, and more specifically for Indian adolescents. In this regard, our recommendation is more on the side of increasing awareness on the benefits of sleep on the cognitive and non-cognitive skills development at the societal level, and to push for more research in this area.

## Summary and conclusion

This paper investigates the association of parental investment on the cognitive and non-cognitive skills development of adolescents living in India. We take an adolescent’s time spent in school, studying, sleeping, and playing, as indicators of parental investment decisions relying on evidence in related literature that parental control on children continues even at the adolescent stage [11, 17]. We measure cognitive skills by looking at the Peabody Picture Vocabulary test (PPVT) score and create factor scores to proxy latent non-cognitive skills, namely resilience and self-esteem. Our results show that all time use variables (i.e., sleeping, playing, studying and at school), after correcting for endogeneity issue, have a positive association on cognitive skills (i.e., PPVT score). While all-time use variables have a positive association on resilience, for self-esteem we find negative associations with time spent playing, studying and at school. In this regard, our results have two important implications.

When it comes to cognitive skills, while we might expect that only time spent in school and study would be positively associated with cognitive skills; in this study, we find that the time spent playing and sleeping is also positively associated with cognitive skills development. This is equally true for the non-cognitive skills resilience. Therefore, our study highlights the importance of both sleep and play, alongside study and schoolwork, for an adolescent’s skills development. The education literature acknowledges the importance of play, but this discussion has largely centred on early childhood years [56, 79–82]. In contrast, our research shows that play is very relevant even for an adolescent’s development. Moreover, research has also shown that sleep deprivation can negatively affect academic performance among adolescents [76–78], which our study also corroborates not only in the case of cognitive skills but also for non-cognitive skills among adolescents (such as resilience). Currently, most policies at international and national level focus on education and schooling and fail to pay attention to play and sleep.

Second, our results for non-cognitive skills showed that some time use variables have a positive association on resilience, while the same variables have a negative association on self-esteem. We interpret this as pointing to the importance of the quality of time use variables. Therefore, policymakers should introduce policies that improve the quality of time spent of various activities.

We would like to end by acknowledging some of the limitations of our study. Since our study only focuses on the state of Andhra Pradesh, these results can only be generalised to the rest of India with caution. In this regard, future research is needed that can consider other states of India, which will increase the generalizability of our results. Second, in this study because of the high number of missing responses, we did not consider the association between time spent on work and the cognitive/ non-cognitive skills development. However, we feel it is important that future studies do take this interrogation further; because while there is policy agreement that children under the age of eighteen should not work, the minimum legal age in India is still fourteen [83]. Moreover, India still faces high rates of child labour despite the minimum legal age [84]. Consequently, this underscores the importance for a more comprehensive understanding of the likely association of time spent at work by the adolescent on his/her cognitive and non-cognitive skills development. Such an understanding will likely have wider policy implications regarding the time an adolescent should spend at work. Third, while we proxy parental investment via time use variables, we realize that they come with certain data limitations, since we did not have more information on how this time was being spent. For example, for time spent studying did parents actively help their children in doing their homework or not? For time spent sleeping, we do not have more information where does the adolescent sleep? Does he/she have a bed or not? Similarly, for time spent playing we do not know if it is supervised play or not. Moreover, are the adolescents playing within the confinements of their home, or outside? The availability of this information would have added a better understanding regarding the time use variables. In this regard, the Young Lives team can expand this understanding via qualitative methods by undertaking in-depth interviews with both the parents and children to gauge a better understanding on this issue.

Four, our study assumes that parents influence how the offspring spends his/her time. While there is evidence for this in the literature [8–12, 16, 17, 19–22], given that we are dealing with adolescents it is to be expected that adolescents are also exercising control, for example by using influencing strategies on parents [85]. Consequently, parents’ control may only be partial and weak on how they want the adolescent to spend her/his time; and hence our interpretations have to be understood with this caveat in mind. Five, our analysis is limited to only two non-cognitive skills (i.e., resilience and self-esteem). Future research can expand the analysis to other non-cognitive skills (such as self-efficacy, social competency, empathy). Finally, we could only use two rounds in our investigation on adolescents, future research can expand the longitudinal perspective by looking at how cognitive and non-cognitive skills evolve as children transition from adolescence and then to adulthood.

## Supporting information

S1 Table Factor loadings for round two resilience latent variable. (DOCX)

S2 Table Factor loadings for round three resilience latent variable. (DOCX)

S3 Table Factor loadings for round two self-esteem latent variable. (DOCX)

S4 Table Factor loadings for round three self-esteem latent variable. (DOCX)

S5 Table Goodness of fit measure for self-esteem and resilience latent variables. (DOCX)

S6 Table List of controls and instruments used. (DOCX)

S7 Table First difference estimation for self-esteem. (DOCX)

S8 Table First difference estimation for resilience. (DOCX)

S9 Table First difference estimation for Peabody Picture Vocabulary test (PPVT) scores. (DOCX)

S10 Table Instrumental variable estimation for Peabody Picture Vocabulary test (PPVT) scores. (DOCX)

S11 Table First difference estimation for self-esteem with shocks. (DOCX)

S12 Table First difference estimation for resilience with shocks. (DOCX)

S13 Table First difference estimation for PPVT scores with shocks. (DOCX)

S14 Table Instrumental variable estimation for PPVT scores with shocks. (DOCX)

S1 Dataset (XLSX)

S2 Dataset (DTA)

S1 File (DO)
