# Supplementary material for: Adolescent’s time use and skills development: Do cognitive and non-cognitive skills differ?
Source: PLoS One. 2022 Jul 21;17(7):e0271374. doi: 10.1371/journal.pone.0271374 (PMC9302839; doi:10.1371/journal.pone.0271374)
Supplement: S1 Table — (DOCX) [file pone.0271374.s001.docx]

**S1 Table. Factor loadings for round two Resilience latent variable**

|  | | | **Coefficients** | | **Std. Err.** | | | **P>z** | **[95% Conf.** | **Interval]** |
| --- | --- | --- | --- | --- | --- | --- | --- | --- | --- | --- |
|  |  |  | | | | |  |  |  |  |
| Someone to help with problems with studies <-  Latent variable (Resilience round 2) | | |  | |  | | |  |  |  |
|  |  |  | .5330282 | | .1626105 | | | 0.001 | .2143174 | .851739 |
| Constant | | | 10.06008 | | 1.250909 | | | 0.000 | 7.608342 | 12.51181 |
|  | | |  | |  | | |  |  |  |
| Someone to help if worried about something at home <- Latent variable (Resilience round 2) | | |  | |  | | |  |  |  |
|  |  |  | .3529662 | | .1997605 | | | 0.077 | -.0385571 | .7444895 |
| Constant | | | 10.6084 | | .8792287 | | | 0.000 | 8.885143 | 12.33166 |
|  | | |  | |  | | |  |  |  |
| Someone to help if you were being teased by another child <- Latent variable (Resilience round 2) | | |  | |  | | |  |  |  |
|  |  |  | .4187472 | | .1303992 | | | 0.001 | .1631695 | .6743249 |
| Constant | | | 6.611972 | | .4617754 | | | 0.000 | 5.706909 | 7.517035 |
|  | | |  | |  | | |  |  |  |
| Someone to help with advice about religious matter <- Latent variable (Resilience round 2) | | |  | |  | | |  |  |  |
|  |  |  | .4538135 | | .0730031 | | | 0.000 | .3107301 | .5968969 |
| Constant | | | 4.339414 | | .2029036 | | | 0.000 | 3.94173 | 4.737097 |
| Someone to help with getting to school or work <-  Latent variable (Resilience round 2) | | |  |  | |  | |  |  |  |
|  |  |  | .5901097 | | .1216059 | | | 0.000 | .3517666 | .8284529 |
| Constant | | | 7.214078 | | .8245713 | | | 0.000 | 5.597948 | 8.830208 |
