# Supplementary material for: Adolescent’s time use and skills development: Do cognitive and non-cognitive skills differ?
Source: PLoS One. 2022 Jul 21;17(7):e0271374. doi: 10.1371/journal.pone.0271374 (PMC9302839; doi:10.1371/journal.pone.0271374)
Supplement: S2 Table — (DOCX) [file pone.0271374.s002.docx]

**S2 Table. Factor loadings for round three Resilience latent variable**

|  | **Coefficients** | **Std. Err.** | **P>z** | **[95% Conf. Interval]** | |
| --- | --- | --- | --- | --- | --- |
| Is there someone who would help - if you were having problems at work? <-  Latent variable (Resilience round 3) |  |  |  |  |  |
|  | .3516827 | .1247853 | 0.005 | .107108 | .5962573 |
| Constant | 6.336706 | .3441759 | 0.000 | 5.662134 | 7.011279 |
|  |  |  |  |  |  |
| Is there someone who would help - If you were worried about something at home? <-  Latent variable (Resilience round 3) |  |  |  |  |  |
|  | .9032842 | .0368173 | 0.000 | .8311236 | .9754449 |
| Constant | 4.614289 | .8552127 | 0.000 | 2.938103 | 6.290476 |
|  |  |  |  |  |  |
| Is there someone who would help - If you were being teased or bullied by another <-  Latent variable (Resilience round 3) |  |  |  |  |  |
|  | .3986542 | .0888123 | 0.000 | .2245852 | .5727231 |
| Constant | 2.867776 | .1388308 | 0.000 | 2.595672 | 3.139879 |
