# Supplementary material for: Adolescent’s time use and skills development: Do cognitive and non-cognitive skills differ?
Source: PLoS One. 2022 Jul 21;17(7):e0271374. doi: 10.1371/journal.pone.0271374 (PMC9302839; doi:10.1371/journal.pone.0271374)
Supplement: S3 Table — (DOCX) [file pone.0271374.s003.docx]

**S3 Table. Factor loadings for round two Self-esteem latent variable**

|  | **Coefficients** | **Standard Errors** | **P>z** | **[95% Conf. Interval]** | | | |
| --- | --- | --- | --- | --- | --- | --- | --- |
|  |  |  |  |  |  | | |
| I feel proud to show my friends where I live <- Latent variable (Self-esteem round 2 |  |  |  |  |  | | |
|  | .1322453 | .0532133 | 0.013 | .0279491 | .2365415 | | |
| Constant | 6.517342 | .438306 | 0.000 | 5.658278 | 7.376406 | | |
|  |  |  |  |  |  | | |
|  |  |  |  |  |  | | |
| I am ashamed of my clothes <- Latent variable (Self-esteem round 2) | -.4108083 | .0728618 | 0.000 | -.5536148 | -.2680018 | | |
| Constant | 1.807721 | .068306 | 0.000 | 1.673844 | 1.941599 | | |
|  |  |  |  |  |  | | |
|  |  |  |  |  |  | | |
| I am often embarrassed because I do not have the right supplies for school <- Latent variable (Self-esteem round 2) | -.4812573 | .0612727 | 0.000 | -.6013495 | -.361165 | | |
| Constant | 1.545019 | .0207923 | 0.000 | 1.504267 | 1.585772 | | |
|  |  |  |  |  |  | | |
|  |  |  |  |  |  | | |
| I am worried that I don’t have the correct uniform <- Latent variable (Self-esteem round 2) | -.7600844 | .0821778 | 0.000 | -.9211499 | -.5990188 | | |
| Constant | 1.606104 | .0372824 | 0.000 | 1.533032 | 1.679176 | | |
|  |  |  |  |  |  | | |
|  |  |  |  |  |  |  |  |
