# Supplementary material for: Adolescent’s time use and skills development: Do cognitive and non-cognitive skills differ?
Source: PLoS One. 2022 Jul 21;17(7):e0271374. doi: 10.1371/journal.pone.0271374 (PMC9302839; doi:10.1371/journal.pone.0271374)
Supplement: S4 Table — (DOCX) [file pone.0271374.s004.docx]

**S4 Table. Factor loadings for round three Self-esteem latent variable**

|  | **Coefficient** | **Std. Err.** | **P>z** | **[95% Conf. Interval]** | |
| --- | --- | --- | --- | --- | --- |
| I am proud of my shoes/ chappals or of having shoes/ chappals <- Latent variable (Self-esteem round 3) |  |  |  |  |  |
|  | .694271 | .0388383 | 0.000 | .6181493 | .7703927 |
| Constant | 3.293665 | .1130248 | 0.000 | 3.07214 | 3.51519 |
|  |  |  |  |  |  |
| I am proud of my clothes <- Latent variable (Self-esteem round 3) |  |  |  |  |  |
|  | .9376004 | .0257815 | 0.000 | .8870695 | .9881312 |
| Constant | 3.564137 | .1294375 | 0.000 | 3.310444 | 3.81783 |
|  |  |  |  |  |  |
| I am proud that I have the correct uniform <- Latent variable (Self-esteem round 3) |  |  |  |  |  |
|  | .5643553 | .048755 | 0.000 | .4687972 | .6599133 |
| Constant | 3.62205 | .1533289 | 0.000 | 3.321531 | 3.92257 |
|  |  |  |  |  |  |
| I feel my clothing is right for all occasions <- Latent variable (Self-esteem round 3) |  |  |  |  |  |
|  | .5223318 | .0443562 | 0.000 | .4353952 | .6092684 |
| Constant | 5.140334 | .2477195 | 0.000 | 4.654813 | 5.625856 |
|  |  |  |  |  |  |
