# Supplementary material for: Adolescent’s time use and skills development: Do cognitive and non-cognitive skills differ?
Source: PLoS One. 2022 Jul 21;17(7):e0271374. doi: 10.1371/journal.pone.0271374 (PMC9302839; doi:10.1371/journal.pone.0271374)
Supplement: S5 Table — (DOCX) [file pone.0271374.s005.docx]

**S5 Table. Goodness of fit measure for Self-esteem and Resilience latent variables**

| **Latent Variable** | **Round** | **Comparative Fit Index*** |
| --- | --- | --- |
| Self-esteem | Round two | 1 |
|  | Round three | 0.972 |
| Resilience | Round two | 1 |
|  | Round three | 1 |
|  |  |  |

*Comparative fit index above 0.9 is considered as an acceptable model fit (Hu & Bentler, 1999).

**Reference:**

Hu, L. T., & Bentler, P. M. (1999). Cutoff criteria for fit indexes in covariance structure analysis: Conventional criteria versus new alternatives. Structural equation modeling: a multidisciplinary journal, 6(1), 1-55.
