# Supplementary material for: Adolescent’s time use and skills development: Do cognitive and non-cognitive skills differ?
Source: PLoS One. 2022 Jul 21;17(7):e0271374. doi: 10.1371/journal.pone.0271374 (PMC9302839; doi:10.1371/journal.pone.0271374)
Supplement: S6 Table — (DOCX) [file pone.0271374.s006.docx]

**S6 Table. List of controls and instruments used**

| **Variables** | **Description of the variable** | **Included in the first difference estimation** | **Included in the Instrumental variable estimation** |
| --- | --- | --- | --- |
| Round | Dummy variable, 1= Round three and 0=Round two | X | X |
| Travel time to school | Time travelling to school in minutes | *Not included* | X |
| Problems with reading | 1= Reading problems | *Not included* | X |
| **Regional variables** | | | |
| Regions | Two regional dummy variables were included. 1= coastal region and 1= Rayalseema | X | X |
| Urban | A dummy variable where 1= Urban | *Not included because it is time invariant* | X |
| **Parental variables** | | | |
| Father’s age | Age in years | X | X |
| Mother’s age | Age in years | X | X |
| Father’s education | Educational level in years | *Not included because it is time invariant* | X |
| Mother’s education | Educational level in years | *Not included because it is time invariant* | X |
| Parental attitude | Arithmetic mean of parent’s attitude | *Not included because it is time invariant* | X |
| **Adolescent variables** | | | |
| School type | Dummy variable, 1= Public school | X | X |
| Grade level | Highest grade level | X | X |
| Health | Dummy variable for health, 1= no malnutrition | X | X |
| Gender | Dummy variable, 1= Male | *Not included because it is time invariant* | X |
| Religion | Dummy variable, 1= Hindu | *Not included because it is time invariant* | X |
| Ethnicity | Dummy variable, 1= Schedule caste tribe | *Not included because it is time invariant* | X |
| **Household variables** | | | |
| Part of a program | Two dummy variables, 1= part of the caste-based welfare program, 1= part of the National Rural Employment Guarantee Scheme. | X | X |
| Wealth | Used the wealth index variable | X | X |
| Household size | Two variables were included 1) Household size that is number of people in the house 2) The number of children in the house | X | X |
