# Supplementary material for: Adolescent’s time use and skills development: Do cognitive and non-cognitive skills differ?
Source: PLoS One. 2022 Jul 21;17(7):e0271374. doi: 10.1371/journal.pone.0271374 (PMC9302839; doi:10.1371/journal.pone.0271374)
Supplement: S10 Table — (DOCX) [file pone.0271374.s010.docx]

**S10 Table. Instrumental variable estimation for Peabody Picture Vocabulary test (PPVT) scores**

| **Percentile PPVT Score** | **Coefficient** | **Robust Std. Err.** | **P>z** | **[95% Conf. Interval]** | | | | |
| --- | --- | --- | --- | --- | --- | --- | --- | --- |
|  |  |  |  |  | |  | | |
| Time spent playing | 19.88266 | 6.888834 | 0.004 | 6.380794 | | 33.38453 | | |
| Time spent studying | 19.46104 | 8.4682 | 0.022 | 2.863673 | | 36.05841 | | |
| Father’s age | -.1362976 | .3936962 | 0.729 | -.907928 | | .6353328 | | |
| Mother’s age | .1384208 | .4748214 | 0.771 | -.792212 | | 1.069054 | | |
| Father’s education level | .1056324 | .3492465 | 0.762 | -.5788782 | | .7901429 | | |
| Mother’s education Level | .3691096 | .516611 | 0.475 | -.6434294 | | 1.381649 | | |
| Regional1, Coastal=1 | 4.036037 | 4.407275 | 0.360 | -4.602064 | | 12.67414 | | |
| Regional2, Rayalaseema=1 | 15.68651 | 6.573887 | 0.017 | 2.801933 | | 28.5711 | | |
| School type, public=1 | -4.001782 | 3.46547 | 0.248 | -10.79398 | | 2.790414 | | |
| Child’s highest grade | 4.915639 | 1.441948 | 0.001 | 2.089473 | | 7.741805 | | |
| Round | -30.10959 | 5.350697 | 0.000 | -40.59677 | | -19.62242 | | |
| No malnutrition | 2.339672 | 2.811951 | 0.405 | -3.171651 | | 7.850996 | | |
| Wealth Index | 14.00738 | 8.725539 | 0.108 | -3.094362 | | 31.10912 | | |
| Male=1 | -6.112568 | 5.997189 | 0.308 | -17.86684 | | 5.641707 | | |
| Hindu=1 | 2.019404 | 3.633666 | 0.578 | -5.10245 | | 9.141258 | | |
| Schedule caste tribe=1 | -.6195605 | 3.056425 | 0.839 | -6.610044 | | 5.370923 | | |
| Part of the National Rural Employment Guarantee Scheme=1 | -3.563812 | 3.021717 | 0.238 | -9.486269 | | 2.358645 | | |
| Part of the caste-based welfare program=1 | -1.053308 | 4.627372 | 0.820 | -10.12279 | | 8.016174 | | |
| Time spent sleeping | 11.26334 | 4.889798 | 0.021 | 1.679515 | | 20.84717 | | |
| Time spent in school | 18.66507 | 6.133515 | 0.002 | 6.643604 | | 30.68654 | | |
| Constant | -337.0081 | 118.6989 | 0.005 | -569.6536 | | -104.3626 | | |
|  |  |  |  |  |  | |  |  |
