# Supplementary material for: Adolescent’s time use and skills development: Do cognitive and non-cognitive skills differ?
Source: PLoS One. 2022 Jul 21;17(7):e0271374. doi: 10.1371/journal.pone.0271374 (PMC9302839; doi:10.1371/journal.pone.0271374)
Supplement: S12 Table — (DOCX) [file pone.0271374.s012.docx]

**S12 Table. First difference estimation for Resilience with shocks**

| **Variables** | **Explanation** |
| --- | --- |
| RCINDEX_factor | Resilience is the non-cognitive variable created from confirmatory factor analysis |
| dadage | Father’s age |
| momage | Mother’s age |
| coastal | Region1, Coastal=1 |
| rayalaseema | Region2, Rayalaseema=1 |
| public | School type, public=1 |
| chgrade | Child’s highest grade |
| round | Round |
| nomalnutrition | No malnutrition |
| hhsize | Household size |
| numbchild | Number of children in the household |
| wi | Wealth index |
| inregegs | Part of the National Rural Employment Guarantee Scheme |
| inwelfar | Part of the caste-based welfare program |
| time_spent_sleeping | Time spent sleeping |
| time_spent_inschool | Time spent in school |
| time_spent_studying | Time spent studying |
| time_spent_playing | Time spent playing |
| shfam5 | Shock1-illness of mother |
| shfam4 | Shock 2- illness of father |
| _cons | Constant |
