# Supplementary material for: Adolescent’s time use and skills development: Do cognitive and non-cognitive skills differ?
Source: PLoS One. 2022 Jul 21;17(7):e0271374. doi: 10.1371/journal.pone.0271374 (PMC9302839; doi:10.1371/journal.pone.0271374)
Supplement: S13 Table — (DOCX) [file pone.0271374.s013.docx]

**S13 Table. First difference estimation for PPVT scores with shocks**

| **Percentile PPVT score** | **Coefficient** | **Robust Std. Errors** | **P>t** | **[95% Conf.**  **Interval]** | |
| --- | --- | --- | --- | --- | --- |
| Father’s age | 4.587504 | 1.017719 | 0.000 | 2.457395 | 6.717614 |
| Mother’s age | 3.032185 | 2.215143 | 0.187 | -1.604163 | 7.668532 |
| Region1, Coastal=1 | -12.7333 | 5.626492 | 0.036 | -24.50969 | -.9569187 |
| Region2, Rayalaseema=1 | 34.07601 | 7.253897 | 0.000 | 18.89343 | 49.25859 |
| School type, Public=1 | -2.103898 | 3.250562 | 0.525 | -8.907401 | 4.699606 |
| Child’s highest grade | 3.072146 | 2.020447 | 0.145 | -1.156698 | 7.30099 |
| Round | -31.04417 | 12.12844 | 0.019 | -56.42929 | -5.659055 |
| No malnutrition | 1.864842 | 2.683744 | 0.496 | -3.752298 | 7.481982 |
| Household size | .9119365 | 1.459042 | 0.539 | -2.141874 | 3.965746 |
| Number of children in the household | .2522685 | 2.248469 | 0.912 | -4.453831 | 4.958368 |
| Wealth Index | 23.12453 | 11.44305 | 0.058 | -.8260543 | 47.07512 |
| Part of the National Rural Employment Guarantee Scheme=1 | -7.715845 | 6.037689 | 0.217 | -20.35287 | 4.921184 |
| Part of the caste-based welfare program=1 | 3.375372 | 3.876884 | 0.395 | -4.73904 | 11.48978 |
| Time spent sleeping | 2.618965 | 1.204081 | 0.042 | .0987954 | 5.139136 |
| Time spent in school | 1.380046 | 1.102217 | 0.226 | -.9269206 | 3.687012 |
| Time spent studying | 3.08149 | 1.354287 | 0.035 | .2469346 | 5.916045 |
| Time spent playing | 2.657288 | .8021216 | 0.004 | .9784282 | 4.336148 |
| Shock1, illness of mother | .2184569 | 3.076642 | 0.944 | -6.221028 | 6.657942 |
| Shock2, illness of father | -1.377423 | 3.699668 | 0.714 | -9.120916 | 6.366071 |
| Constant | -322.4395 | 100.3356 | 0.005 | -532.4443 | -112.4347 |
|  |  |  |  |  |  |
