# Supplementary material for: Adolescent’s time use and skills development: Do cognitive and non-cognitive skills differ?
Source: PLoS One. 2022 Jul 21;17(7):e0271374. doi: 10.1371/journal.pone.0271374 (PMC9302839; doi:10.1371/journal.pone.0271374)
Supplement: S14 Table — (DOCX) [file pone.0271374.s014.docx]

**S14 Table. Instrumental Variable estimation for PPVT scores with shocks**

| **Percentile PPVT score** | | **Coefficient** | | **Robust Std. Err.** | | **P>z** | **[95% Conf. Interval]** | | | | |
| --- | --- | --- | --- | --- | --- | --- | --- | --- | --- | --- | --- |
|  |  | |  | |  |  |  |  |  |  |  |
| Time spent playing | | 20.00231 | | 6.908338 | | 0.004 | 6.462219 | | 33.54241 | | |
| Time spent studying | | 19.78523 | | 8.507662 | | 0.020 | 3.110515 | | 36.45994 | | |
| Father’s age | | -.1326639 | | .4019097 | | 0.741 | -.9203924 | | .6550646 | | |
| Mother’s age | | .1334051 | | .4832592 | | 0.783 | -.8137656 | | 1.080576 | | |
| Father’s educational level | | .1029712 | | .3518708 | | 0.770 | -.5866829 | | .7926253 | | |
| Mother’s educational level | | .3618774 | | .5163345 | | 0.483 | -.6501196 | | 1.373874 | | |
| Region1, Coastal=1 | | 3.968261 | | 4.433598 | | 0.371 | -4.721431 | | 12.65795 | | |
| Region2, Rayalaseema=1 | | 15.71067 | | 6.589035 | | 0.017 | 2.796402 | | 28.62495 | | |
| School type, public=1 | | -3.992124 | | 3.481928 | | 0.252 | -10.81658 | | 2.83233 | | |
| Child’s highest grade | | 4.90301 | | 1.449277 | | 0.001 | 2.062479 | | 7.74354 | | |
| Round | | -30.16842 | | 5.357263 | | 0.000 | -40.66846 | | -19.66838 | | |
| No malnutrition | | 2.361935 | | 2.823483 | | 0.403 | -3.171991 | | 7.89586 | | |
| Wealth Index | | 13.9371 | | 8.792787 | | 0.113 | -3.296446 | | 31.17064 | | |
| Male=1 | | -6.218844 | | 6.003602 | | 0.300 | -17.98569 | | 5.548 | | |
| Hindu=1 | | 2.015042 | | 3.673795 | | 0.583 | -5.185463 | | 9.215547 | | |
| Schedule caste tribe=1 | | -.6321281 | | 3.079519 | | 0.837 | -6.667875 | | 5.403619 | | |
| Part of the National Rural Employment Guarantee Scheme=1 | | -3.588956 | | 3.038716 | | 0.238 | -9.54473 | | 2.366817 | | |
| Part of the caste-based welfare program=1 | | -1.186561 | | 4.624784 | | 0.798 | -10.25097 | | 7.87785 | | |
| Time spent sleeping | | 11.39865 | | 4.907147 | | 0.020 | 1.780817 | | 21.01648 | | |
| Time spent in school | | 18.832 | | 6.165595 | | 0.002 | 6.747654 | | 30.91634 | | |
| Shock1, illness of mother | | 1.196817 | | 4.543408 | | 0.792 | -7.708099 | | 10.10173 | | |
| Shock2, illness of father | | -.3165193 | | 4.366947 | | 0.942 | -8.875577 | | 8.242539 | | |
| Constant | | -340.4017 | | 119.2608 | | 0.004 | -574.1486 | | -106.6549 | | |
|  | |  | |  | |  |  |  | |  |  |
